# Flexibility of neural circuits regulating mating behaviors in mice and flies

**DOI:** 10.3389/fncir.2022.949781

**Published:** 2022-11-08

**Authors:** Tomomi Karigo, David Deutsch

**Affiliations:** ^1^Kennedy Krieger Institute, Baltimore, MD, United States; ^2^The Solomon H. Snyder Department of Neuroscience, Johns Hopkins University School of Medicine, Baltimore, MD, United States; ^3^Sagol Department of Neurobiology, Faculty of Natural Sciences, University of Haifa, Haifa, Israel

**Keywords:** sexual behavior, reproduction, internal states, sexual maturation, social experience, sexual satiety, persistent states, neuromodulation

## Abstract

Mating is essential for the reproduction of animal species. As mating behaviors are high-risk and energy-consuming processes, it is critical for animals to make adaptive mating decisions. This includes not only finding a suitable mate, but also adapting mating behaviors to the animal’s needs and environmental conditions. Internal needs include physical states (e.g., hunger) and emotional states (e.g., fear), while external conditions include both social cues (e.g., the existence of predators or rivals) and non-social factors (e.g., food availability). With recent advances in behavioral neuroscience, we are now beginning to understand the neural basis of mating behaviors, particularly in genetic model organisms such as mice and flies. However, how internal and external factors are integrated by the nervous system to enable adaptive mating-related decision-making in a state- and context-dependent manner is less well understood. In this article, we review recent knowledge regarding the neural basis of flexible mating behaviors from studies of flies and mice. By contrasting the knowledge derived from these two evolutionarily distant model organisms, we discuss potential conserved and divergent neural mechanisms involved in the control of flexible mating behaviors in invertebrate and vertebrate brains.

## Introduction

**FIGURE 1:**
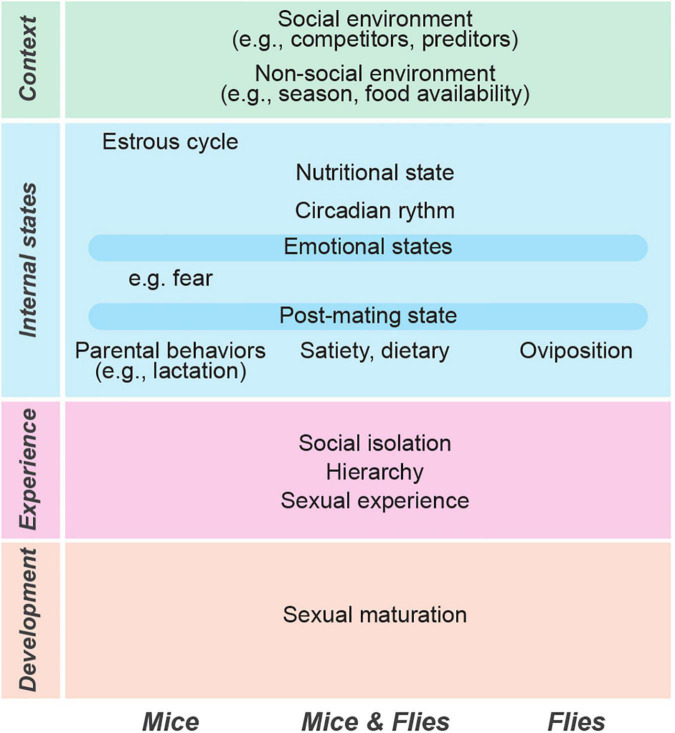
Multiple factors drive flexible mating behaviors in mice and flies. The common factors that affect mating behaviors in both species are listed in the center. The unique factors that are known to affect only one species are shown in the left (mice) and right (flies).

Reproductive behaviors are sometimes considered to be pre-programmed, “instinctive,” or “innate,” meaning that they are controlled by genetically hardwired circuits and not dependent on previous experience (Tinbergen, 1951). While they clearly have a hardwired component, innate behaviors, including mating behaviors, must also adapt in response to changing needs, environmental conditions, and the animal’s own history. Indeed, ethological studies in multiple organisms have demonstrated the flexibility of mating behaviors across taxa, with mating decisions being dependent on both intrinsic and extrinsic factors (Figure 1). Intrinsic factors include sexual maturation (Prevot, 2015; Zhang et al., 2021a), reproductive state (Lynch et al., 2005; Phillips-Farfán and Fernández-Guasti, 2009; Zhou et al., 2014), nutritional state (Jones and Wade, 2002; Grosjean et al., 2011; Lebreton et al., 2015), circadian rhythms (Sakai and Ishida, 2001; Miller and Takahashi, 2013), sleep (Lesku et al., 2012; Chen et al., 2017), and age (Forslund and Pärt, 1995; Prosser et al., 1997; Moore and Moore, 2001; Brenman-Suttner et al., 2020). Extrinsic factors reflect both social and non-social environments, including the availability and quality of prospective mates and rivals (Jirotkul, 1999; Preston and Stockley, 2006; Bretman et al., 2011), the risk of predation (Rick and Dill, 1993; Godin and Briggs, 1996; Jirotkul, 1999), ambient light (Gamble et al., 2003), seasonal changes (Borg et al., 2006; Milner et al., 2010), temperature (Gayou, 1984; Schnebel and Grossfield, 1984; Wilson, 2005; Conrad et al., 2017), and food availability (Marsteller and Lynch, 1987; Harshman et al., 1988; Billeter and Wolfner, 2018; Ando et al., 2020).

Intrinsic and extrinsic factors often interact with one another. For example, evidence of an available food source may drive a hungry individual to favor foraging and feeding over mating, whereas food availability may enhance courtship or receptivity in a non-hungry individual. Intrinsic factors can also be periodic (e.g., estrous cycle) or reflect certain prior experiences, including both recent (e.g., a failed courtship attempt or a recent stressful event) and more distant events (e.g., previous mating, aggressive encounters, lack of food, or a formed association between some environmental cue and behavioral outcomes).

When exploring the behavioral processes that govern mating-related decision-making, several key questions arise: What are the neural mechanisms underlying flexible mating behaviors? Which brain circuits enable this flexibility? How flexible are the circuits that control mating behaviors in healthy and unhealthy individuals? Flexibility in mating or post-mating behaviors can also arise at different levels in the sensory-motor axis. For example, female flies change their taste preferences post-mating (Ribeiro and Dickson, 2010; Walker et al., 2015), and a surge of dopamine (DA) in the main olfactory bulb of the female mouse shortly after mating impairs the perception of social odors present in male urine, which is likely to protect against miscarriage (Serguera et al., 2008). In these examples, post-mating gustatory or olfactory responses are modified in the females to improve the odds of successful fertilization by ensuring adequate nutrient availability and avoiding mating competition with other sexual partners. Motor control of mating behaviors is also dependent on the mating state. For example, in *Drosophila melanogaster*, females extend their ovipositor to reject a courting male (Kimura et al., 2015) through the activation of DNp13 (or “pMN1”) projection neurons (Wang et al., 2020a). Ovulation, triggered by prior mating, enhances DNp13 motor output (Wang et al., 2020a). In mice, a longer duration of intromission relative to mounting has been observed among sexually experienced males (Swaney et al., 2012).

Substantial progress has been made in recent decades with respect to the understanding of the neural circuits underlying mating behaviors in mice and flies (Auer and Benton, 2016; Lenschow and Lima, 2020), and emerging studies are beginning to reveal how these circuits are modulated by internal and external factors in both species.

### The control of mating behaviors in mice

Mating decisions depend on the integration of multiple sensory cues over time. When a mouse encounters an opposite-sex conspecific, it will display a chain of stereotypic mating behaviors. During this encounter, if males and females are sexually receptive, they display sexual appetitive behaviors. A male mouse will follow and pursue a female and investigate his potential mate, particularly in the anogenital area. A female mouse will indicate sexual motivation by repeatedly approaching the male and engaging in darting movements (Pfaus et al., 2015). During the initial interaction phase, males and females begin to vocally communicate through ultrasonic vocalizations (USVs) (for a review, see Egnor and Seagraves, 2016). Males actively vocalize toward females during courtship, and male USVs increase when males approach females (Pomerantz et al., 1983; Hammerschmidt et al., 2009; Asaba et al., 2017), but females also emit USVs during courtship (Hammerschmidt et al., 2012; Neunuebel et al., 2015), although this has not been studied to the same degree as male courtship vocalization. Vocal communication will continue throughout the social interactions, however, USV syllable types vary depending on the interaction phase, indicating that USVs may play different roles in particular phases of the courtship process (Matsumoto and Okanoya, 2016). Once a male has successfully pursued and motivated a female, he will attempt to mount her from the rear, grasping her flanks with his forepaws and displaying pelvic thrusting movement. If a female is sexually receptive, she will present receptive behaviors including lordosis, which involves curving the lumbar region of the back toward the floor. If a female is not receptive, she will display rejection behaviors, including escaping, kicking, and a defensive posture to avoid the male’s mating attempts, often emitting audible broadband vocalizations (squeaks) when the male attempts to mount. Once a male has successfully mounted a receptive female, he will proceed to intromission with reduced thrusting speed and deeper movement, which is visibly distinguishable from the thrusting performed during mounting. A variable number of mounts and intromissions will be performed until reaching ejaculation. After a male has completed copulation through ejaculation, he will engage in the post-copulatory grooming of his genital area. After ejaculation, the male enters a refractory period in which he is not attracted by the same receptive female for at least 24 h (McGill, 1962). However, a male will sometimes copulate with a new female after just 2 or 3 h of rest (McGill, 1962, 1963). This will be discussed further in section “Recent mating experience (sexual satiety).” The mating behaviors of the house mice (*Mus musculus*) have been extensively described in the literature (King, 1956; McGill, 1962; Latham and Mason, 2004).

Mice are nocturnal and normally perform mating under complete darkness, suggesting they rely less on visual information for mating. Olfaction, on the other hand, is critical for mediating mating behaviors in mice. The main olfactory system detects volatile odorants through the main olfactory epithelium (MOE) and is critical in both males and females for the establishment of appropriate social interactions, as MOE ablation and the genetic ablation of olfactory signaling *via* knocking out cyclic nucleotide-gated channel a2 (CNGA2) results in decreased sexual behaviors in both sexes (Mandiyan et al., 2005; Keller et al., 2006a,b; Matsuo et al., 2015). Another family of odorants—pheromones—is detected both through the vomeronasal organ (VNO) and the MOE, modulating the behavior or physiology of conspecific individuals. VNO ablation leads to deficient mating behaviors in both males and females (Bean, 1982; Clancy et al., 1984; Keller et al., 2006b), and the genetic ablation of pheromonal signal transduction *via* knocking out transient receptor potential cation channel c2 (TRPC2) alters sex-specific social behaviors (Leypold et al., 2002; Stowers et al., 2002; Kimchi et al., 2007; Yu, 2015). Thus, both the vomeronasal and the main olfactory systems are necessary for the regulation of mating behavior in both males and females. Somatosensory cues also contribute to the control of mating behavior and are particularly well-studied in the context of sexual receptivity in female rats. The application of mechanical stimuli to the female flank, perineum, and base of the tail promotes lordosis in female rats in the absence of males (Kow et al., 1979). Throughout investigation and mating, male and female mice contact one another with their paws, noses, and whiskers. Social somatosensory stimuli during courtship may also affect sexual receptivity and motivation in mice.

**FIGURE 2:**
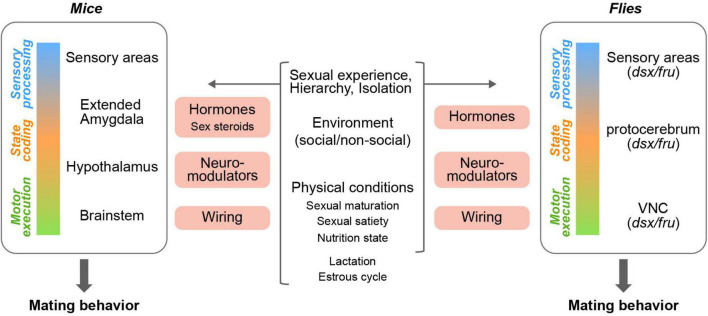
Circuit nodes in mating neural circuits that are influenced by internal and external factors. Diverse internal and external factors (middle) affect mating neural circuits through various mediators (pink boxes) at different levels (left and right) in both mice and flies.

The neural circuits that control mating behavior in mice have been investigated for over decades. Studies utilizing brain lesioning, electric stimulation, and immediate early gene labeling in rodents have suggested that the limbic system, extended amygdala, and hypothalamus are the crucial brain structures governing sexual behaviors in males and females (Figure 2; Vaughan and Fisher, 1962; Larsson and Heimer, 1964; Pfaff and Sakuma, 1979; Pfaus and Heeb, 1997; Newman, 1999; Pfaff et al., 2006), many of which are enriched in the expression of sex steroid receptors (Simerly et al., 1990; Lauber et al., 1991; Shughrue et al., 1992, 1997; Shughrue and Merchenthaler, 2001; Mitra et al., 2003; Zuloaga et al., 2014). Recent advances in genetic tools and systems neuroscience approaches have enabled us to further dissect these neural circuits. In this review, however, we will not focus on the detailed neural circuits controlling mating behaviors (for reviews, see Anderson, 2016; Hashikawa et al., 2016; Chen and Hong, 2018), but will rather explore the internal and external factors that modulate mating behaviors, sexual motivation, and mate choice decisions.

### The control of mating behaviors in flies

The mating rituals of fruit flies (*Drosophila melanogaster*) have been studied extensively since they were first described in detail over a century ago (Sturtevant, 1915). Male flies undergo a stereotyped courtship ritual that includes following the female, tapping her, playing a courtship song with extended wings, licking her genitalia, and finally mounting (Dickson, 2008). The ultimate decision to mate or not to mate is made by the female (Zhou et al., 2014; Wang et al., 2020a). While often described as a linear process, this courtship ritual is in fact complex and highly dynamic. For example, the male may chase and sing between multiple copulation attempts, and song characteristics vary greatly between singing epochs (Clemens et al., 2015), in part owing to the fact that male singing is modulated by dynamic sensory cues from the female (Coen et al., 2014; Calhoun et al., 2019). As the female speed depends on the male song (Coen et al., 2014, 2016), male song and female locomotion concurrently modulate one another. This courtship ritual is not only dynamic but also multisensory. Males use visual, olfactory, and gustatory cues when chasing and evaluating the female, while the female responds to visual, olfactory, and auditory cues from the courting male (or males). Much of the circuitry that controls mating behavior in *Drosophila melanogaster* has been dissected in recent years, as reviewed by others (see Yamamoto and Koganezawa, 2013; Auer and Benton, 2016). Importantly, neurons that express the sex determination genes *fruitless (fru)* and *doublesex (dsx)* were found to play a pivotal role in controlling fly mating behaviors (Figure 2; Ryner et al., 1996; Greenspan and Ferveur, 2000; Cachero et al., 2010; Yu et al., 2010; Yamamoto and Koganezawa, 2013). For example, some sexually dimorphic neurons have been shown to be tuned to specific aspects of the male song (Baker et al., 2019; Deutsch et al., 2019), while other neurons drive male singing or female receptivity (von Philipsborn et al., 2011; Zhou et al., 2014). Although more is known regarding the underlying circuitry in males, the neural basis of female mating behaviors is also beginning to emerge, and it has become evident that females take an active role in the mating process. Depending on her mating status, the female may exhibit active rejecting behaviors, changing her speed in response to the male song or extruding her ovipositor to signal her willingness to mate (Rezával et al., 2012; Feng et al., 2014; Kerwin and von Philipsborn, 2020; Mezzera et al., 2020; Wang et al., 2020a), and possibly even singing during copulation (Kerwin et al., 2020), although the specific role of this song is currently unknown (Kerwin and von Philipsborn, 2020). Recent advances in the fine automatic quantification of mating behaviors (Pereira et al., 2020), and in tracing neural circuits at a synaptic level using EM-based connectomes (Scheffer et al., 2020; Dorkenwald et al., 2022) are expected to accelerate the dissection of mating circuits in both sexes.

## Flexible circuits for mating behaviors in mice and flies

While much progress has been made in deciphering the circuits underlying mating behaviors in mice and flies, we know far less about the mechanisms through which these circuits are flexibly modulated as a function of internal and external factors. Internal factors include developmental modifications, periodic changes (e.g., estrous cycle), and other physiological changes that reflect changing needs (e.g., hunger state). External factors include environmental cues, both social and non-social. It is important to keep in mind that internal and external factors often interact. For example, the effect of smelling male bedding (external) depends on the female’s estrous state (internal) (Dey et al., 2015), and both light and temperature (external) affect the circadian phase (internal) that in turn modulates mating behaviors both in mice and in flies (Sakai and Ishida, 2001; Vitaterna et al., 2001). We suggest a layered view of the factors that modulate mating (Figure 1). Developmental changes (sexual maturation) are shaped by previous experience (social and non-social), by the current internal state of the animal (e.g., mating motivation or nutritional state), and by the context of the mating episode (the immediate social and non-social environment).

Sexual maturation depends mostly on internal (developmental) factors; social hierarchy, social isolation, social experience, and recent mating experience depend on interactions with the external social environment, but can also be affected by internal factors. For example, how a previous fight affects mating behaviors in the present depends on both the past interactions with the environment (e.g., opponents) and on the current state of the animal (e.g., receptivity or fear state). Environmental context refers to the modulation of mating behaviors by social and non-social factors in the immediate surroundings, thus depending primarily on external factors. Food-sex interactions include both the animals’ nutritional state (internal factor) and food availability (external factor). Nutritional state and food availability interact such that a hungry fly or mouse is more likely to prioritize food intake over mating when food is available.

In the following sections we discuss these factors individually in mice and flies, keeping the following questions in mind:

1. Which nodes in the circuitry that controls mating behaviors are modulated, and by which factors?
2. Which mechanisms allow for flexibility in the control of mating behaviors over short and long timescales?
3. Which mechanisms for flexible mating behaviors are shared between mice and flies, and which are not?

### Sexual maturation

Mating is a costly process in terms of energy consumption, competition over other needs (such as feeding or sleeping), external risks (such as predation or competition with rivals), and physiological costs (such as disease transmission, injury, or reduced lifespan) (Daly, 1978; Wigby and Chapman, 2005; Baer et al., 2006; Emery Thompson and Georgiev, 2014). Therefore, it is important that mating occur only when the energy balance is favorable for reproduction, and when physiological and behavioral risks are low. Specifically, mating behaviors should match the maturation of the reproductive organs in both sexes. Here we discuss sexual behavioral maturation in male and female mice and flies.

#### Sexual maturation and mating behaviors in mice

As mammals enter into puberty, factors including somatic growth, energy balance, and the season begin to activate the Hypothalamus-Pituitary-Gonadal (HPG) axis, the central pathway that regulates the reproductive system. In both males and females, pubertal onset is triggered by the activation of hypothalamic circuitry, which ultimately controls the reawakening of gonadotropin-releasing hormone (GnRH) neurons over the course of pubertal development. Among these circuits, kisspeptin neurons are important players that activate GnRH neurons to promote the further maturation of the HPG axis (d’Anglemont de Tassigny et al., 2007; Prevot, 2015). High-frequency GnRH secretion leads to gonadotropin release from the pituitary, which leads to gametogenesis and an increase in gonadal steroid hormone secretion from ovaries and testes. Steroid hormones, such as estrogens and androgens, activate and remodel the adolescent brain, leading to the development of sexual behavior, including the salience of sensory stimuli, sexual motivation, and the execution of mating behaviors.

Sexual maturation can be measured by the physical properties of reproductive organs. In female mice, puberty can be determined by vaginal opening, which occurs around postnatal day (PND) 26–30, or by the first estrus as assessed by cytological analysis of vaginal smears, which occurs around PND 35–40 (Oboti et al., 2017). In male mice, puberty can be determined by preputial separation around PND 26–30 (Mayer et al., 2010; Novaira et al., 2014; Hoffmann, 2018). In males, serum testosterone levels start to increase around PND 35 and rise to adult levels at around PND 45 (Jean-Faucher et al., 1978; Wu et al., 2010; Wang J. Y. et al., 2015). During mid-adolescence (PND 35–47), mice become fully fertile with the elevated secretion of gonadal steroid hormones (Jean-Faucher et al., 1978). However, sexual maturity precedes behavioral maturity. In male rodents, reproductive behavior typically emerges 1–2 weeks after the onset of the pubertal rise in testosterone secretion. In females, the display of behavioral estrus cycles lags behind vaginal opening by a similar amount of time (Williams and Scott, 1954; Södersten et al., 1977). Sexual behavioral maturation during puberty depends on the timing of gonadal maturation and increases in serum sex steroid hormone levels, as steroid hormones are required to induce reproductive behavior. However, studies have shown that behavioral maturation is not solely driven by the presence of steroid hormones at puberty. Hormone treatment in prepubertal animals fails to fully activate sexual behavior in male and female rodents (Baum, 1972; Olster and Blaustein, 1989; Schulz et al., 2004), indicating that the presence of steroid hormones is insufficient and that maturation in the neural circuit and peripheral organs are necessary for prepubertal animals to activate these behaviors. In male hamsters, prepubertal castration reduces testosterone-induced activation of sexual behavior in adulthood compared to castration after puberty. Neither prolonged hormone replacement nor sexual experience in adulthood reverses these behavioral deficits (Schulz et al., 2004, 2009). This suggests that sex steroid-induced neural circuit reorganization during puberty is a critical and irreversible mediator of behavioral maturation.

In addition to the maturation of the animal displaying sexual behavior, the maturation of the sexual partner is also important, as sexual behavior toward juvenile animals is inhibited in adult males and females through sensory stimuli. A juvenile pheromone, exocrine-gland secreting peptide 22 (ESP22), is produced by young mice. ESP22 is secreted from the lacrimal gland and released into tears of 2–3 week-old mice. ESP22 is sensed by the VNO and strongly inhibits adult male sexual behavior toward juvenile mice (Ferrero et al., 2013) and suppresses sexual receptivity in adult female mice (Osakada et al., 2018). Such mechanisms enable mice to avoid immature mates and enhance their reproductive success.

#### Sexual maturation and mating behaviors in flies

There are four morphologically distinct stages of *Drosophila* development: embryo, larva (three instar stages), pupa, and adult. Progression through all of these stages is dictated by pulses of the steroid hormone 20-hydroxyecdysone (20E) (Thummel, 2001).

Both males and females are sexually immature for the first days after eclosion (Spieth, 1974; McGill and Manning, 1976). Male courtship intensity is gradually elevated to a maximum courtship intensity 72 h after eclosion in *Drosophila melanogaster* (Zhang et al., 2021a), and female receptivity (measured as the percent of copulated females) saturates at 48 h post-eclosion in *Drosophila simulans* and *melanogaster* (Manning, 1967). Sexually immature adult females reject male courtship by running or jumping away, kicking, and fluttering their wings (Connolly and Cook, 1973). Some rejecting behaviors are unique to immature females, while others are shared with old or recently mated unreceptive females (Aranha and Vasconcelos, 2018).

Several studies have sought to clarify the degree to which the timing of behavioral sexual maturation is correlated with body growth and the maturation of sex organs. Male sperm length is highly variable across *Drosophila* species, and flies with longer sperm tend to also have a prolonged adult non-reproductive phase during which they do not copulate with virgin females, indicating a correlation, over evolutionary timescales, between physiological and behavioral sexual maturation (Pitnick et al., 1995). More broadly, genetic studies of *Drosophila melanogaster* have shown that the central regulatory pathways, including hormonal signaling, that control growth and the timing of sexual maturation (McBrayer et al., 2007) are conserved through evolution, suggesting that these aspects of animal life history are regulated by a common genetic architecture (Tennessen and Thummel, 2011).

The cellular and molecular mechanisms underlying developmental changes during the embryonic, larval, and pupal stages have been subject to intensive research (see Cagan and Ready, 1989; Riddiford and Ashburner, 1991; Campos-Ortega and Hartenstein, 2013). Much less is known about the mechanisms underlying sexual maturation during adulthood. It has been shown that behavioral sexual maturation is enabled through a decay in the levels of Juvenile hormone (JH) in both males (Wijesekera et al., 2016; Zhang et al., 2021a) and females (Manning, 1967; Ringo et al., 1991), though little is known regarding the specific effects of JH (and potentially other signals) on the maturation of specific nodes in the mating-control circuitry in either sex. In *Drosophila*, JH is synthesized *de novo* in a specialized endocrine gland, the corpus allatum (CA). DA controls JH levels by either stimulating or inhibiting its synthesis and degradation depending on the developmental stage (Gruntenko et al., 2012). JH regulates multiple processes in *Drosophila* development and lifespan. This “hormonal pleiotropy” has been posited to synchronize multiple aspects of animal life, possibly balancing tradeoffs between competing needs (Flatt et al., 2005).

There is conflicting evidence as to whether JH suppresses sexual maturation by inhibiting the sensory response to other-sex cues. Male courtship intensity and the sensitivity of Or47b olfactory receptor neurons (ORNs), which promote courtship by detecting the aphrodisiac pheromone palmitoleic acid (Lin et al., 2016), also increases with age and peaks at about 1 week (Kvelland, 1965; Lin et al., 2016). Males lacking the Or47b receptor exhibit reduced wing extension frequency and are outcompeted by wild-type males in their efforts to court females (Dweck et al., 2015; Lin et al., 2016). It has been argued that the coordination of Or47b neuronal sensitivity with male fertility depends on JH. Through its interaction with a putative JH receptor (Wilson and Fabian, 1986; Jindra et al., 2013), JH modulates the sensitivity of Or47b ORNs, thus elevating male courtship vigor. However, under light conditions, males that are mutants for Orb47b court well (Wang L. et al., 2011; Zhang et al., 2021a). In well-lit one-male, one-female courtship assays, Zhang et al. (2021a) observed little or no reduction in mature males’ courtship when the neurons expressing Or47b are silenced and no increase in courtship when these sensory neurons are stimulated in juveniles, therefore arguing that the suppression of sexual behavior in juveniles cannot be explained by adjustments to primary sensory neurons.

Many questions remain open on this topic: Which nodes along the pathways from sensory inputs to motor outputs are controlled by JH? Does JH control all the aspects of sexual maturation, or are there parallel JH-independent pathways? Which aspects of sexual maturation in flies are sex-specific, and which are shared between males and females? Is sexual maturation a gradual or step-like process (Manning, 1967)?

### Social hierarchy

Social hierarchy (dominance hierarchy; social dominance) has been studied for decades (Sidanius and Pratto, 2001) in humans (Sidanius and Pratto, 2001), non-human primates (Cowlishaw and Dunbar, 1991), and other animal species (Squires and Daws, 1975; Portugal et al., 2017), including rodents (Desjardins et al., 1973; Faulkes and Abbott, 1993; Ferreira-Fernandes and Peça, 2022) and insects (Strassmann and Meyer, 1983; Peeters et al., 2000). A dominant higher-ranking individual is sometimes referred to as an “alpha,” while the submissive lower-ranking individual is a “beta.” Different types of interactions can result in dominance depending on the species. In social living groups, members are likely to compete for access to limited resources and mating opportunities. Rather than fighting each time they meet, relative rank is established between individuals of the same sex, with higher-ranking individuals often gaining more access to resources and mates. Based on repetitive interactions, a social order is created that is subject to change each time a dominant animal is challenged by a subordinate one. While the ethology of social hierarchy has been extensively studied, only in recent decades have researchers begun to decipher the underlying mechanisms using genetic model organisms. Here, we focus on the interaction between social hierarchy and mating behaviors in mice and flies.

#### Social hierarchy and mating behaviors in mice

Dominant male mice exhibit higher levels of sexual behavior than subordinate males (Parmigiani et al., 1982a) and dominant males ejaculate more frequently than do subordinates (de Catanzaro and Ngan, 1983). This will result in higher levels of sexual fitness in the dominant males, as suggested by the greater numbers of litters sired by dominant males relative to subordinate males (DeFries and McClearn, 1970; Oakeshott, 1974). Furthermore, estrus females prefer dominant males over subordinate males in a binary choice test (Parmigiani et al., 1982b). Higher sexual performance and attractiveness to females in dominant males can be explained by differences in the hormonal milieu depending on social status. Social subordination suppresses gonadal function in mice (Lombardi and Vandenbergh, 1977; Williamson et al., 2017b), and leads to lower plasma testosterone levels in subordinate males compared to dominant males (Ely, 1981; Machida et al., 1981). Chemical and auditory cues are important for the attractiveness of males to females. Female mice are attracted to male-specific pheromones, such as darcin in male urine (Roberts et al., 2010; Demir et al., 2020). Many male-specific pheromones are synthesized under the control of testosterone (Bronson, 1979) and they are likely diminished in subordinates. Male USVs are attractive to females (Pomerantz et al., 1983; Hammerschmidt et al., 2009; Musolf et al., 2010; Asaba et al., 2017), and the production of USVs is sensitive to sex steroid hormones. Castration-mediated reductions in testosterone levels decrease the number of emitted USVs and subsequent hormone replacement restores such vocalization (Dizinno and Whitney, 1977; Nunez et al., 1978; Nunez and Tan, 1984; Bean et al., 1986). A dominant male emits more USVs toward females than a male of lower social rank (Wang F. et al., 2011). High levels of pheromones and vocalization in a dominant male may make them attractive to females. However, it is important to note that other studies show the relationship between social status and plasma testosterone levels varies across mouse strains and their housing conditions (summarized in Williamson et al., 2017a).

In contrast to males, the relationship between social hierarchy and mating behavior is not clear in females. Although there is no difference in the plasma estradiol level between dominant and subordinate females, subordinate females express higher levels of estrogen receptors (ERα and ERβ) in the ventromedial hypothalamus (VMH) compared to dominant females (Williamson et al., 2019). ERα neurons in the ventrolateral subdivision of VMH (VMHvl) have been suggested to control sexual receptivity and aggression in female mice (Hashikawa et al., 2017; Inoue et al., 2019; Liu et al., 2022). Social hierarchy may alter female sexual behaviors through estrogen-sensitive neurons in the VMHvl, but further studies of this and related topics are needed in females.

#### Social hierarchy and mating behaviors in flies

In *Drosophila melanogaster*, while both males and females compete with same-sex conspecifics for resources (Nilsen et al., 2004), only males establish hierarchical relationships of “winners” and “losers” (Kravitz and Fernandez, 2015). Males that have won previous contests are more likely to win in subsequent conflicts, while losers are more likely to experience recurrent losses (winner-loser effects). Among the male-specific offensive actions, the lunge is particularly important, as its usage predicts the outcome of a fight: the first animal to lunge, if the opponent retreats, is 16 times more likely to be the ultimate winner. Ultimate winners lunge more frequently as fights progress, while losers lunge with decreasing frequency. In second fights, losers are highly unlikely to lunge against familiar opponents and are less likely to lunge against any opponents (Yurkovic et al., 2006).

While the effects of defeat and victory on future male-male aggressive encounters have been studied extensively (Trannoy et al., 2016), much less is known about the role of social hierarchy in modulating mating behaviors in flies, including whether social hierarchy impacts male *Drosophila melanogaster* mating behaviors. Hierarchies established through prior aggressive encounters can impact male mating behaviors through a range of mechanisms. Firstly, winners may be better able to protect more territory, in turn attracting more females (Baxter et al., 2015). Secondly, male winners may have a higher chance of winning a fight against a competing male when fighting for a target female (Baxter et al., 2018). Thirdly, winning or losing fights may impact male courtship displays, thus influencing mating success (Teseo et al., 2016; Filice and Dukas, 2019). A recent study showed that winners have higher pre-copulatory mating success both when paired with and without a naïve competitor male (Filice and Dukas, 2019). Interestingly, these authors also found that losers have a longer copulation duration, resulting in more offspring per copulation (Filice and Dukas, 2019). More work is needed to facilitate the detailed quantification of male courtship behaviors with and without a competitor, and following experiences of winning or defeat.

The neural mechanisms underlying the memory of previous victory or defeat are not well understood. It is evident that winning and losing have both short- and long-term effects, that memory of defeat lasts longer, and that repeated defeats induce a persistent loser effect, which is dependent on *de novo* protein synthesis (Trannoy et al., 2016). Neuromodulators play an important role in controlling aggressive behaviors (Asahina, 2017), and activating a small subset of serotonergic neurons is sufficient to overcome this “loser mentality,” restoring mating motivation in losers (Hu et al., 2020). As courtship and aggression share common neuronal nodes in both males (Hoopfer et al., 2015; Koganezawa et al., 2016) and females (Deutsch et al., 2020; Schretter et al., 2020), it is possible that previous aggressive behaviors impact these common centers, modifying both aggressive and mating drives based on previous experience.

### Social isolation

Social isolation has dramatic consequences for the development of individual members of many vertebrate and invertebrate species. Such isolation induces a set of behavioral disturbances including the ability to appropriately process environmental and social stimuli, while also contributing to increased activity/arousal, aggression, and, in some cases, social avoidance. While the importance of social interaction for healthy development is well recognized, the underlying mechanisms are not well understood.

#### Social isolation and mating behaviors in mice

Social isolation is a factor that promotes stress-mediated neural and endocrine changes in social animals such as mice and rats, and can have prolonged and profound effects on various social and non-social behaviors (Zelikowsky et al., 2018a; Lee et al., 2021). Extended social isolation enhances aggressive behaviors in male mice (Zelikowsky et al., 2018b), although the effects of social isolation on sexual behaviors are less well understood. Exposure to social isolation stress, especially during puberty, can lead to long-term behavioral alteration in adulthood. In rats, post-weaning social isolation leads to a reduction of sexual behaviors in both males and females (Duffy and Hendricks, 1973; Spevak et al., 1973). In male mice, post-weaning social isolation decreases sexual preference toward females in a three-chamber assay and reduces mating behaviors (Liu et al., 2019). In female mice, prolonged social isolation during puberty lead to reduced sexual receptivity in adulthood, and re-socialization in adulthood is insufficient to rescue the effects. In Females that have undergone such isolation exhibit altered hypothalamic ERα expression, suggesting that social isolation during puberty period may affect sex steroid hormone-dependent brain remodeling and reorganization during this period (Kercmar et al., 2014).

Social isolation during adulthood yields distinct outcomes. For example, the exposure of adult male mice to social isolation for periods from 1 h to 2 weeks increases both appetitive and consummatory sexual behaviors (de Catanzaro and Gorzalka, 1980). Notably, this same 2-week social isolation treatment results in reduced sexual performance in male rats and gerbils, but has no major impact on male hamsters, suggesting the effects of social isolation on reproductive behaviors are species-dependent across rodents (de Catanzaro and Gorzalka, 1979).

#### Social isolation and mating behaviors in flies

Studies of various *Drosophila* species have shown that even closely related species can have profoundly differing responses to social isolation (Chen and Sokolowski, 2022). In *Drosophila melanogaster*, social isolation enhances aggression, whereas social grouping reduces aggression (Hoffmann, 1990; Wang et al., 2008). Whether and how social isolation modulates mating behaviors, however, is less well understood.

Consistent with the idea that males with previous social experiences have an advantage in finding a mating partner, it has been reported that in a competitive assay, males who were reared in a social environment have a marked advantage in courting females when competing against males who were reared in isolation (Sethi et al., 2019). Researchers have found that group housing enhances the responsiveness of specific Or47b neurons. High Or47b neuron activity initiates a signaling event that enhances the efficacy of JH (Wijesekera et al., 2016; Zhang et al., 2021a), suggesting a possible mechanism that links social-housing experience with mating behavior in male flies.

*Drosophila melanogaster* males that harbor mutations in the sex determination gene *fru* do not court unless they are raised with other flies (Pan and Baker, 2014). Interestingly, group-reared (but not singly-housed) *fru*-null males have been found to initiate and maintain courtship-like behaviors following the pursuit of a horizontally moving light spot, even without the priming of the courtship command neurons (“pC1” and “pC2”) (Kohatsu and Yamamoto, 2015). These two findings suggest that social experience enhances courtship behaviors and can even overcome the effects of genetic mutation.

Social isolation also impacts the neural and behavioral responses of males and females to courtship songs. *Drosophila* males and females are innately tuned to conspecific courtship song parameters (Cowling and Burnet, 1981; Arthur et al., 2013). This auditory tuning likely contributes, along with olfactory cues, to the isolation barrier between sub-species of *Drosophila* (Ewing and Bennet-Clark, 1968; Cowling and Burnet, 1981; Legendre et al., 2008). The group housing of males, but not females, has been found to sharpen the tuning to the interpulse interval (IPI; a conspecific characteristic of *Drosophila* courtship song) both behaviorally through a change in walking speed in response to pulse-song playback and neuronally at the activity level of the pulse-detecting pC2 cells. As sharper tuning in group-housed flies has been observed in males but not in females, this led researchers to posit that the sex-specific sharpening arises from the fact that group-housed males (but not females) are exposed to courtship song from other flies (Deutsch et al., 2019). Consistently, playback-mediated conspecific song exposure in young flies can enhance behavioral responses to conspecific IPI in both sexes (Li et al., 2018).

There is limited evidence that male song differs between group-housed and socially isolated males. Marie-Orleach et al. (2019) found that males produce longer song bouts during courtship when reared with social partners as compared to males that were reared in isolation, though the reported effect was relatively small. Mean bout duration of male song is correlated with female speed (Clemens et al., 2015), and the change in female speed in response to male song is associated with female receptivity (Coen et al., 2014). It is possible that males who produce longer song bouts exhibit an advantage when courting a receptive female, but such a causal link has yet to be shown.

Social isolation can also have indirect effects on male courtship success through its effect on other social phenotypes. First, socially isolated males present with higher levels of aggression (Wang et al., 2008), and fighting experience may influence mating behavior (Filice and Dukas, 2019). Second, socially isolated and group-housed flies form distinct social networks, potentially influencing their mating decisions in the presence of other flies (Bentzur et al., 2021). Third, social isolation may lead to greater access to nutritional resources such that socially isolated males may be larger and therefore more successful in mating (Kim and Ehrman, 1998). Chronic social isolation was found to modulate the expression of multiple genes, including several linked to feeding behaviors (Li et al., 2021). For example, the expression of *Drosulfakinin* (DSK), which was previously demonstrated to be involved in modulating male sexual behavior (Wu et al., 2019), decreases twofold after chronic isolation (Li et al., 2021).

While accumulating evidence suggests that social isolation affects mating behaviors in male flies, more work is needed to reveal mechanisms underlying the effect of social isolation on mating behaviors in both sexes.

### Social experience

Sexual behaviors are strongly modulated by the prior experiences of the animals and by the associated social context (see Hirsch and Tompkins, 1994; Wei et al., 2021; Chen and Sokolowski, 2022). These effects can be acute or long-lasting, affecting mating behaviors at multiple levels including sensory detection, motivation to mate, and motor performance. Here, we focus on the effects of sexual experience on mating behaviors in mice and flies.

#### Social experience and mating behaviors in mice

Sexual experience is an important modifier of mating behaviors in mice, having a long-term impact on both the appetitive (approach and investigate mate) and consummatory (motor behaviors) components of mating behaviors. Mating behavior is governed by a complex interaction between different systems in the brain which process sensory inputs, regulate reward and motivation, integrate hormonal signals, and control copulation movement itself. All of these components are impacted by sexual experience, and sexual experience generally enhances the performance of sexual behaviors in both males and females.

In male rodents, the detection of female odor is a critical trigger for the initiation of male sexual behavior. While disruption of either the olfactory or vomeronasal system has severe effects on male sexual behavior in many rodents (Powers and Winans, 1975; Steel and Keverne, 1985; Mandiyan et al., 2005), these effects are less severe if subjects are sexually experienced when they undergo disruptive manipulation (Meredith, 1986; Pfeiffer and Johnston, 1994). Furthermore, sexually experienced male mice acquire preferences for the odors of receptive females with sexual experience (Hayashi and Kimura, 1974). This suggests that sexual experience alters the neural circuitry responsible for detecting female odor in rodents. Sexual experience also influences the motor control of sexual behaviors. Studies in different rodent species, including mice (Swaney et al., 2012), rats (Larsson, 1959; Dewsbury, 1969), and guinea pigs (Valenstein et al., 1955), have shown that behavioral components of copulation, including mounting, intromission, and ejaculation occur with shorter latencies and higher frequencies in sexually experienced males. Gonadal steroids, such as estrogens and androgens, play an essential role in regulating sexual behaviors, as castration-mediated reductions in sex steroid production suppress sexual behavior while subsequent testosterone treatment restores this behavior (McGill and Manning, 1976; McGinnis and Dreifuss, 1989). However, animals that undergo castration after sexual experience tend to retain their ability to display sexual behaviors to a greater degree than do individuals castrated without sexual experience in both mice (Manning and Thompson, 1976) and hamsters (Costantini et al., 2007). This suggests that sexual experiences modify the neural circuit that integrates steroid hormone signals with behavioral output. The secretion of testosterone itself is also affected by sexual experience. Circulating testosterone levels increase during a sexual encounter after exposure to female cues and during courtship (Batty, 1978a,b; Amstislavskaya and Popova, 2004; Nyby, 2008; Gleason and Marler, 2010). Sexual experience enhances both of these female-triggered reflexive releases of testosterone (Kamel et al., 1975; Bonilla-Jaime et al., 2006) and increases baseline levels of circulating testosterone (Edinger and Frye, 2007; Wu and Gore, 2009). In addition, the expression of androgen receptor (AR) is increased in the medial preoptic area (MPOA) in sexually experienced mice (Swaney et al., 2012), which is noteworthy as this is a critical brain region for the control of male sexual behavior (Bean et al., 1981; Wei et al., 2018; Karigo et al., 2021). It has also been reported that the density of mature dendritic spines in the MPOA is increased in sexually experienced males (Jean et al., 2017). Altogether, elevated gonadal steroid levels, higher levels of hormonal sensitivity, and increased plasticity in the mating behavior circuit ultimately contribute to higher sexual performance in sexually experienced males.

In female rodents, the performance of sexual behavior is often assessed based on the interest shown in response to male cues (appetitive) and the lordosis reflex (consummatory). Together with exogenous hormone treatment, sexually experienced females show an enhanced preference for male urine over female urine compared to naïve females (McCarthy et al., 2018). Expression of synaptophysin (SYP), a presynaptic vesicle marker, in the AOB but not in the MOB, increases in sexually experienced females as compared to sexually naïve females (Marco-Manclus et al., 2022). This suggests that plasticity in the AOB triggered by sexual experience may increase preference for male cues in experienced females. Sexually naïve female mice do not display high levels of sexual receptivity in their first sexual experience; they require several sexual encounters to display the full receptive response (Edwards, 1970; Laroche et al., 2009a,b; Bonthuis et al., 2011; Ismail et al., 2011). The plasticity in the reward circuit may play a vital role in increased receptivity in sexually experienced females. The mesolimbic reward circuit is characterized by dopaminergic (DAergic) projections from the ventral tegmental area (VTA) to nucleus accumbens (NAc) and the activity in this pathway is known to encode reward predictions and facilitate reinforcement learning (Watabe-Uchida et al., 2017). Numbers of studies have reported the importance of the this pathway in sexual motivation. Elevated DA release in the NAc upon encounters with opposite-sex conspecifics and during mating has been observed in both male and female rats (Mas et al., 1990; Wenkstern et al., 1993; Mermelstein and Becker, 1995; Pfaus et al., 1995) and mice (Goto et al., 2015; Sun et al., 2018; Dai et al., 2021). Similarly, in female hamsters, DA levels are elevated in the NAc during sexual interactions (Meisel et al., 1993), and females with multiple prior sexual interactions present with augmented DA release relative to that in inexperienced females (Kohlert and Meisel, 1999). Furthermore, sexual experience leads to a morphological change in the mesolimbic system. Sexual experience increases dendritic spine density in medium spiny neurons (MSNs) of female hamsters, doing so selectively in excitatory D1 receptor-expressing MSNs in the core of the NAc (Staffend et al., 2014). Together, the DAergic system may mediate sexual motivation in females. Sexual experience enhances DA release and neuronal plasticity in the NAc and increases the motivational components of female sexual behavior, manifesting in higher levels of receptivity in experienced females.

#### Social experience and mating behaviors in flies

Mating behaviors in *Drosophila* are largely innate. For example, male flies do not require tutoring in order to sing a courtship song (Arthur et al., 2013), and females are innately tuned to multiple conspecific features of the courtship song (Deutsch et al., 2019). The best-studied example of the effects of learning on *Drosophila* mating behavior is “courtship conditioning,” which refers to an association that male flies form between a sex pheromone (cVA) and the mating status of the female (Ejima et al., 2007; Keleman et al., 2012). The sex pheromone cVA is produced only by male flies and is transferred to females during copulation (Everaerts et al., 2010). Therefore, recently mated females carry cVA. Males that have previously courted and been rejected by a mated female learn to associate the smell of cVA with rejection, and will therefore avoid courting mated females in the future (Siegel and Hall, 1979). Some of the underlying circuitry for this association has been revealed (Keleman et al., 2012; Zhou et al., 2012; Montague and Baker, 2016), and it has been shown that neuromodulators (in particular DA and Octopamine; Keleman et al., 2012; Zhou et al., 2012) play important roles in such “courtship conditioning.”

Strikingly, a recent study revealed that mating experience also modulates the wiring of olfactory neurons in the female brain (Chou et al., 2022). A set of inhibitory olfactory local neurons (“TC-LN”) exhibit specific and significantly increased innervation of the VL2a glomerulus, possibly contributing to feeding behaviors in post-mated females. As VL2a neurons are postsynaptic to Ir84 ORNs, whose activation strongly modulates male courtship behavior (Grosjean et al., 2011), this also raises the possibility that the experience-dependent modulation of VL2a wiring also governs mating behaviors in male and female flies.

Fly mating behaviors are clearly modulated by social experience at multiple levels, from auditory and olfactory perception to the association between sensory cues and aversive stimuli (mating rejection), to motor control (song production). However, many open questions related to this topic remain: How is male courtship ritual modulated by previous social experience? Does premature mating modulate female adult mating behavior (Connolly and Cook, 1973; Markow, 2000)? Is there a critical period for the social experience-dependent modulation of mating behaviors in male and female flies? Answering these questions at the behavioral level in *Drosophila* species will provide a foundation for a better understanding of the cellular and molecular mechanisms through which social experience shapes the nervous systems under ethologically relevant scenarios.

### Recent mating experience (sexual satiety)

Remating is valuable for the propagation of a species if the probability of having more or better offspring is higher following remating. The value of repeated mating depends on the specific reproductive system of each organism and sex. The degree to which the amount of male ejaculation or the fertilization success of females changes as a function of the recent mating history differs between species. There is an adaptive value in having a lower sexual motivation (or “sexual satiety”) for repeated mating if the value of repeated mating is low, or if the cost of repeated mating is high, and this has spurred research interest in the circuits and molecular mechanisms that control sexual satiety in males and females.

#### Sexual satiety and mating behaviors in mice

Male sexual behavior is strongly inhibited by sexual satiety. Male sexual satiety or exhaust occurs as a result of repeated copulation with the same receptive female, during which several ejaculatory series are achieved. This state of sexual satiety and a concomitant lack of interest in females lasts for an extended period, requiring between 3 and 15 days for the full recovery of sexual drive in rats (Rodríguez-Manzo and Fernández-Guasti, 1994, 1995b; Rodriguez-Manzo, 1999a; Phillips-Farfán and Fernández-Guasti, 2009) and several days in mice (McGill, 1963; Zhang et al., 2021b). Male sexual satiety is caused by reduced sexual motivation due to repeated ejaculations, but is not a consequence of fatigue or motor inability (Phillips-Farfán and Fernández-Guasti, 2009). A series of studies in rats have shown that this state of sexual satiety can be reversed through behavioral or pharmacological manipulations that are capable of re-establishing sexual motivation. DAergic transmission at the mesolimbic system plays a central role in regulating male sexual motivation. Pharmacological activation of DAergic signaling can reactivate copulation in sexually satiated male rats (Rodríguez-Manzo, 1999b). A recent study has shown that activation of DA release from the anteroventral periventricular nucleus (AVPV) and preoptic periventricular nucleus (PVpo) to the MPOA re-activates sexual motivation in recently satiated male mice (Zhang et al., 2021b). Sexual motivation can also be restored in satiated males by replacing the mated female with a new receptive female. This phenomenon is known as the “Coolidge effect” (Fisher, 1962; Wilson et al., 1963; Brown, 1974; Dewsbury, 1981; Rodríguez-Manzo, 1999b; Tlachi-López et al., 2012). In these studies, once males had reached sexual satiety with a female after multiple ejaculations, a novel female or the same female was introduced into the male cage. The percentage of males that achieved ejaculation with the second female was higher when a new female was introduced than following the re-introduction of the same female. Satiated males can exhibit the motor ejaculatory behavior to newly introduced females, even though the size of the seminal plug is significantly reduced relative to that associated with pre-satiated ejaculation, and no sperm is detected in the resultant seminal plug (Phillips-Farfán and Fernández-Guasti, 2009; Tlachi-López et al., 2012; Lucio et al., 2014). The number of intromissions required for the first ejaculation with the second female was higher than the first female (Fisher, 1962), suggesting that these males have not fully recovered from the previous mating session. Many studies have examined the Coolidge effect in rates, but the utilized behavioral paradigms varied slightly among studies and the effect size varied substantially. It has been hypothesized that the Coolidge effect is caused by an increase in sexual motivation due to the presence of the new female, which serves a sexually incentivizing stimulus. The introduction of novel females increases DA concentrations in the NAc in satiated males (Fiorino et al., 1997). Sexual satiety is also affected by serotoninergic signaling. The inhibitory effect of serotonin on male sexual behavior (5-HT) has been shown in pharmacological studies in rats (Bitran and Hull, 1987; Fernández-Guasti et al., 1992). Serotonin concentration increases in the lateral hypothalamic area (LHA) after ejaculation, and infusion of serotonin in LHA reduces DA concentration in NAc (Lorrain et al., 1997, 1999). Furthermore, opioid agonists inhibit male mating behavior (McIntosh et al., 1980; Pfaus and Gorzalka, 1987). In satiated males, the opioidergic system is activated across the brain, and particularly long-lasting activation of this system has been observed in the hypothalamus (Rodríguez-Manzo et al., 2002). Treatment with opioid antagonists prevents or shortens the refractory period in male rats (Koskinen et al., 1991; Rodríguez-Manzo and Fernández-Guasti, 1995a).

The neuroendocrine system is also involved in the control of male sexual satiety. Although plasma testosterone and estradiol levels are similar between non-mated and sexually satiated male rats at 24 h post-mating, AR density was reduced in the MPOA, VMH, and NAc (Fernandez-Guasti et al., 2003), and the density of ERα was increased in the MPOA, medial amygdala (MeA), lateral septum (LS), and NAc, but decreased in the bed nucleus of the stria terminalis (BNST) in sexually satiated males (Phillips-Farfán and Fernández-Guasti, 2007). These data suggest that sexual satiety may partly be caused by altered sensitivity to sex steroid hormones.

Relative to males, the mechanisms governing female rodent sexual satiety have been less well studied. Paced mating paradigms are often used to measure a female rodent’s sexual motivation and desire. This is accomplished using pacing chambers composed of two chambers separated by a divider with one or more small holes that only the female can pass through (Erskine, 1985; Erskine et al., 1989; Paredes and Alonso, 1997; Paredes and Vazquez, 1999). The male is enclosed in one side of the chamber and the female is free to pace the interaction with the male by choosing a side of the chamber. Both female rats and mice (Johansen et al., 2008; Farmer et al., 2014) display similar paced mating behavior. In a conditional place preference (CPP) test, female rats develop a preference for the mating chamber when mating was paced, but no preference was found when mating was not paced by females (Oldenburger et al., 1992; Paredes and Vazquez, 1999; Marco-Manclus et al., 2022), suggesting that mating is rewarding when a female is able to control the initiation and rate of copulation freely without being paced to mate by a male. To induce sexual receptivity in female rodents, ovariectomy (OVX) plus estrogen and progesterone treatment models is are often used to mimic estrus by controlling ovarian steroid hormone levels. OVX plus hormone-treated receptive female rats exhibit low levels of sexual receptivity (lordosis) 12 h after mating only when they were allowed to pace mating during the first mating session (Erskine and Baum, 1982), suggesting that sexual satiety occurs only when females can control mating and that it does not simply depend on the sensory stimuli that females receive.

In a paced mating paradigm, both in rats and mice, the pattern of approach toward and withdrawal from the male is dependent on prior interactions. Females are more likely to leave the chamber with the male as the intensity of the preceding male mating behavior increases (mount without intromission < intromission < ejaculation). The latency to return to the male chamber also depends on the preceding stimulation, with the return latency being longer following an ejaculation than following a mount or an intromission (Erskine, 1985, 1989; Johansen et al., 2008). These data suggest that females differentiate between the varying intensities of copulatory stimuli, and that they control the temporal sequence of the copulatory stimuli which they receive from males. Whether the prolonged return latencies seen after a large amount of intromission reflect increasing sexual satiety or increased aversion to excessive irritation in the genital area during intromission under these circumstances is not known.

Much as in males, monoamines and opioids have been reported to inhibit lordosis or potentially related to sexual satiety in female rats. The activation of delta opioid receptors in the MPOA inhibits lordosis (Sinchak et al., 2004), and the activation of mu opioid receptors in the VMH inhibits lordosis (Acosta-Martinez and Etgen, 2002). At 12 h after mating, higher serotonin and serotonin metabolite levels have been observed in the brain stem of females who received intromission (Erskine and Baum, 1982). Increased 5-HT neuron activity impairs lordosis behavior, whereas decreased activity has the opposite effect (Meyerson, 1964; Yamanouchi et al., 1982). The application of a monoamine oxidase inhibitor around the VMHvl reduces lordosis in female rats (Luine and Fischette, 1982). However, these prior studies have focused on the effects of these neurotransmitters on lordosis, but not specifically on sexual satiety. The relationship between sexual satiety in females and these neurotransmitters remains unclear, and further research is necessary. In addition, mating alters sensory perceptions in female mice. The scent of the urine of an unfamiliar male, but not that of the male they mated with, blocks embryo implantation between days 0 and 3 of pregnancy (Bruce effect) (Bruce, 1959). One to three days after mating, DA levels in the main olfactory bulb surge, and neurons in the olfactory bulbs of recently mated female mice become less sensitive to male urine pheromones. This leads females to be less interested in male urine and contributes to the maintenance of pregnancy (Serguera et al., 2008).

#### Sexual satiety and mating behaviors in flies

Male and female flies copulate multiple times in both laboratory and natural settings (Harshman et al., 1988; Markow, 1988; Gromko and Markow, 1993; Singh et al., 2002), raising the question of how prior encounters modulate sexual satiety in males and females. The male’s motivation to court a female declines after copulation. This effect is mediated at least in part through the role of DAergic neurons in the anterior superior medial protocerebrum (SMPa). This DA SMPa signal is sensed by P1 cells in the male’s central brain (Zhang et al., 2016). Two recent studies suggest that recurrent connectivity between excitatory cells allows for brain activity underlying a persistent state of mating motivation in males (Zhang et al., 2019; Jung et al., 2020). This loop involves the *dsx*-expressing pCd neurons and a set of neuropeptide F (NPF)-expressing cells. The NPF-pCd recurrent loop is also connected to the P1 neurons. Interestingly, this courtship circuit mechanism appears to be under homeostatic control, as copulation-reporting neurons (CRNs) in the abdominal ganglion suppress the NPF signaling to the DA SMPa neurons, reducing the mating drive. The CREB2-dependent transcription of specific K + channels (TASK7) in the NPF-pCd recurrent loop leads to persistent satiety following mating. Another aspect of male mating motivation relates to the drive of a given male to maintain copulation for multiple minutes despite competing stimuli. Males retain copulation motivation even when facing a threatening stimulus. This motivation slowly decays down to a low level around 6 min after the initiation of copulation, which corresponds to the time it takes to transfer the sperm to the female. This effect depends on a slow decay of CaMKII kinase activity in the courtship circuit (Thornquist et al., 2020).

A female’s mating motivation (or receptivity) also depends on her recent mating history. A mated female shows rejecting behaviors toward a courting male (Connolly and Cook, 1973; Cook and Connolly, 1973; Kimura et al., 2015; Aranha and Vasconcelos, 2018; Wang et al., 2020a). This effect is partially mediated through the role of a male seminal fluid peptide (sex peptide) that is injected into the female abdomen with the male seminal fluid during copulation (Feng et al., 2014; Wang et al., 2020a). Artificial injection of sex peptide to a virgin female is sufficient to make her unreceptive, and to reverse her response to male courtship song (Chen et al., 1988; Coen et al., 2014). Notably, sex peptide also drives oocyte maturation in sexually mature adult females by regulating the downstream levels of JH (Soller et al., 1999).

A receptive female signals her willingness to mate by opening her vaginal plates, allowing the male to copulate. Vaginal plate opening (VPO) occurs in response to the male courtship song and is dependent on the mating status of the female. Therefore VPO depends on the integration of both exteroceptive (male courtship song) and interoceptive (mating status) inputs (Wang et al., 2021). The sex peptide that is injected into the female with the male seminal fluid is detected by sex peptide sensory neurons (SPSNs). Sex peptide downregulates the excitability of the SPSNs, and hence their input onto their target—the ascending “SAG” neurons that relay the signal to the *Dsx*-expressing pC1a neurons in the female central brain (Feng et al., 2014; Wang et al., 2020b). Through this cascade, the female mating state (recent mating) regulates her receptivity. VPO descending neurons (vpoDNs) integrate the female mating status, through the pC1 cells, with excitatory input from auditory neurons (vpoENs) to drive female VPO. Interestingly, the SAG, vpoDN, and pC1 neurons all express the sex determination transcription factor *Dsx* (*Doublesex*). Taken together, this suggests that subsets of the sexually dimorphic pC1 cells control mating motivation in both sexes (P1 in males, pC1a in females), and are involved in mediating reduced mating motivation following recent copulation. Recurrent connectivity between pC1 cells and other groups seems to play a role in controlling persistent mating motivation in males, and in controlling a persistent aggressive state in females (Deutsch et al., 2020; Wang et al., 2021). Whether recurrent connectivity is also involved in driving a persistent mating motivation in females is still unknown. While there is some evidence for the role of DA in the modulation of pre-mating sexual motivation in females (Ishimoto and Kamikouchi, 2020), it remains unclear what DA plays in controlling sexual satiety in females, and to what degree the mechanisms governing DA-dependent sexual satiety are similar in males and females.

Beyond the role of the sex peptide, it has also been shown that the sensory experience of copulation is sufficient to induce a reduction in female receptivity. Neurons expressing the mechanosensory channel Piezo transmit the sensory detection of copulation to a pair of ascending LSAN neurons, which relay the information to Myoinhibitory peptide (MIP)-expressing neurons in the female brain (Shao et al., 2019). On the male side, ejaculation itself is rewarding. Repeated activation of the Crz neurons that drive ejaculation is also rewarding to the male (Zer-Krispil et al., 2018). This reward drives an increase in the levels of NPF (the fly homolog of neuropeptide Y) in the fly brain (Zer-Krispil et al., 2018). How the act of copulation (ejaculation in males, sensory experience of copulation in females) contributes to lower mating motivation following recent copulation is still to be revealed.

### Environmental context

Both social cues (e.g., the existence of predators or competitors) and non-social cues (e.g., temperature or humidity) in the immediate environment can modulate mating behaviors. While sexual behavior is often measured in the lab in isolation (a single male and a single female) and under controlled conditions (e.g., similar temperature and humidity across trials), the environmental context has clear ethological relevance for mating decisions. Here, we focus on a few examples that exemplify the impact of such context on mating behaviors in mice and flies.

#### Environmental context and mating behaviors in mice

The social environment influences sexual maturation, reproductive state, and sexual behaviors. In house mice, the sexual maturation of juvenile females is delayed by the presence of group-living adult female mice (Vandenbergh et al., 1972; Drickamer, 1974). In adult females, housing females together in groups causes an irregular estrous cycle or prolonged diestrus phase (Van Der Lee and Boot, 1955; Whitten, 1959; Bronson and Chapman, 1968; Champlin, 1971). The frequency of estrus decreases as the density of females housed together increases. These phenomena affecting reproduction have been proposed to be mediated by a urinary pheromone produced by grouped females (McIntosh and Drickamer, 1977; Jemiolo et al., 1987; Jemiolo and Novotny, 1993; Ma et al., 1998). On the other hand, exposure to males accelerates the onset of puberty in young females and induces synchronized estrous cycles in adult females, and two urinary pheromones found in male urine have been shown to mediate these effects (Whitten, 1956; Novotny et al., 1999).

Exposure to predator-related cues can also alter mating behaviors in males. Prolonged exposure to cat urine, for example, reduces male mating behaviors, likely through vomeronasal signaling (Voznessenskaya, 2014). Predator chemosignals activate the ventromedial subdivision of the VMH (VMHdm) (Ishii et al., 2017), which has been suggested to be involved in defensive behaviors (Silva et al., 2013; Kunwar et al., 2015; Wang L. et al., 2015). Optogenetic activation of VMHdm neurons triggers defensive behaviors such as freezing and jumping, and markedly diminishes ongoing mating behavior in males (Kunwar et al., 2015), suggesting that the neural circuits controlling defensive behaviors and mating behavior are closely linked.

#### Environmental context and mating behaviors in flies

Despite being a non-eusocial insect, the life history and natural habitat of *Drosophila melanogaster* are highly dynamic with respect to their social environment (Reaume and Sokolowski, 2006). Flies aggregate over food patches (Bartelt et al., 1985; Lin et al., 2015), and such convergences are a substrate for a rich repertoire of social interactions that include courtship, competition for mating partners, mating, and communal oviposition (Soto-Yéber et al., 2018). Therefore, social context (such as the number and composition of the surrounding flies) varies between mating events.

Female mating frequency depends on both group composition and size. Females who mate with males of the same strain in the presence of males of other strains have fewer offspring, suggesting a social context-dependent inbreeding avoidance mechanism. Secondly, females mate at a higher frequency in the presence of males from multiple strains, possibly mitigating last male sperm precedence and increase in offspring genetic diversity (Billeter et al., 2012). The mechanisms underlying these context-dependent mating behaviors are still poorly understood. Smell-impaired Orco mutant females do not increase mating frequency according to group composition, indicating that social context-dependent changes in reproductive behavior depend on female olfaction, rather than direct male-male interactions. The effect of strain mixture on mating frequency could be mediated through changes in the regulation of the production of male pheromones under different conditions (Krupp et al., 2008).

More work is needed to reveal how social networks regulate the mating behavior of individual males and females. For example, it remains to be determined as to how surrounding group dynamics (Bentzur et al., 2021) influence male courtship and female responses and choice, and what the underlying neural mechanisms are that are responsible for such modulations. Answering these questions will entail certain technical challenges including tracking the detailed dynamics of courtship behavior (Pereira et al., 2020) while maintaining the identity of individual flies (Gal et al., 2020).

Exposure to predators modulates both mating and post-mating behaviors in females. Parasitoid wasps lay their eggs in the larvae and pupae of certain insect species. When the wasp eggs hatch, they feed on the host insect, eventually killing it (Carton et al., 1986). Exposure to parasitoid wasps leads to a sharp decline in oviposition, likely based on olfactory cues (Ebrahim et al., 2015), and flies exposed to certain wasp species also begin mating more quickly, likely based on visual cues (Ebrahim et al., 2021). The underlying mechanisms may involve the upregulation of some amino acid micropeptides (Ebrahim et al., 2021), but the underlying neural circuit that determined these wasp-induced changes in mating behavior remains largely unknown.

### Food-sex interactions

Feeding and mating are both critical for the survival of any species. While separate circuits control feeding and mating, these two behaviors are not fully independent, prompting research interest in how hunger status (or “starvation-state”) and food availability affect mating behaviors in mice and flies. The impact of food access on mating behaviors depends on a given animal’s hunger state. Food availability may signal a good substrate for egg-laying in flies or the feeding of progeny in mice, as well as a higher probability of a prospective sexual partner being well-fed, therefore enhancing mating probability. On the other hand, starved or recently mated flies may prefer food over sex, in which case food availability may reduce mating probability. Lastly, as mating and post-mating behaviors require energy, this hunger state is expected to influence mating decisions. Below we discuss how hunger and food availability shape mating decisions in mice and flies.

#### Food-sex interactions in mice

Nutritional states have notable impacts on reproductive functions in rodents. Pre-pubertal food restriction leads to delays in puberty onset in both male and female rats (Vandenbergh et al., 1972; Glass et al., 1986; Compagnucci et al., 2002; Tena-Sempere, 2015; Rizzoto et al., 2019). A low energy state caused by food restrictions has a significant impact on the reproductive state in both sexes. Food restrictions lead to a suppression of the HPG axis in both males and females (Wahab et al., 2013; Tena-Sempere, 2015). Male mice that fasted for 9 h have significantly lower blood glucose levels, and a 48-h fast causes a ∼50% reduction in plasma glucose levels (Oosterveer et al., 2008; Jensen et al., 2013). In female rodents, under-feeding reduces serum gonadotropin levels (Howland, 1971; Jones and Wade, 2002), and a 24–48 h fast delays the estrous cycle and suppresses ovulation (Cooper et al., 1970; Bronson and Marsteller, 1985). In males, prolonged food restriction reduces serum gonadotropin levels, reduces testis size, and suppresses spermatogenesis (Glass et al., 1986; Tena-Sempere, 2015).

Nutritional state also affects reproductive functions at the behavioral level. Acute food deprivation or chronic food restriction suppresses receptivity (lordosis) in female rodents (Cooper et al., 1970; Wade et al., 1996; Jones and Wade, 2002). In males, temporal caloric restriction fails to alter sexual behavior, but prolonged food restriction results in a significant decrease in performance and motivation to initiate sexual behavior (Govic et al., 2008; Alvarenga et al., 2009).

Various metabolites that reflect nutrition/hunger state have been suggested to modulate sexual behaviors. Ghrelin is a hormone mainly produced by the stomach that is released at higher levels under conditions of food restriction and promotes food intake (Tschöp et al., 2000). The intracerebroventricular (ICV) injection of ghrelin reduces sexual behavior in males and females (Bertoldi et al., 2011; Babaei-Balderlou and Khazali, 2016). The ICV injection of orexigenic hormone neuropeptide Y (NPY) inhibits sexual behaviors in both male and female rats (Clark et al., 1985), while the injection of anorexigenic hormone alpha-melanocyte-stimulating hormone (α-MSH) and its receptor (MC4R) antagonist increase sexual behavior in female rats and male mice (Van der Ploeg et al., 2002; Pfaus et al., 2004). The injection of α-MSH in the MPOA or VMH increases sexual behaviors in female rats (Gonzalez et al., 1996). Although many studies suggest that metabolic signaling may directly regulate mating behaviors, where and how these feeding-related hormones and neuropeptides act on mating behavior neural circuits is still not clear.

Both mating and feeding are critical behaviors for the survival of the species, raising the question of how behavioral decisions are made when these two conflicting needs coexist. Hunger states change odor preferences. Both male and female mice are equally attracted to food odors and pheromones (opposite sex urine) when they are fed. However, mice investigate food odorants more than pheromones when they have been starved for 24 h. This hunger-dependent food odor attraction is mediated by agouti-related peptide (AGRP)/NPY neurons in the arcuate nucleus (ARC) through the projection to the paraventricular thalamus (PVT) (Horio and Liberles, 2021). A 48-h fasting suppresses sexual behaviors in female mice but not in male mice when food is not presented (Burnett et al., 2019). As hunger strongly drives food intake, a fasted male spends less time engaged in mating when food is presented together with a female. Interestingly, however, when food is absent, a fasted male demonstrates a comparable level of time spent engaging in mating behavior as compared to fed males. The latency to initial mounting is reduced and the number of animals engaged in mating behaviors increased under these fasting conditions. As such, preventing animals from satiating their caloric hunger can tip behavioral choices in favor of satiating reproductive drive, which is the only other satiable motivation at the time (Burnett et al., 2019).

#### Food-sex interactions in flies

*Drosophila melanogaster* are often referred to as “fruit flies” as they (and other closely related species) are associated with the presence of over-ripened fruit and vegetables, where they aggregate, feed, and mate (Spieth, 1974; Lin et al., 2015; Markow, 2015; Soto-Yéber et al., 2018). Therefore, the existence of food and mates near one another has a clear ethological relevance in this species.

A diverse array of neuronal signals induced by hunger and satiety states has been identified in *Drosophila*. Combinations of these signals can be considered representations of hunger states. Most of these signals are neuropeptides, which are modulatory and can potentially mediate the long-range control of multiple neural circuits in the nervous system (Lin et al., 2019). These include, for example, insulin-like peptides and Unpaired 2 (fly equivalents of mammalian insulin and leptin) (Brogiolo et al., 2001), as well as Neuropeptide F (a homolog of mammalian Neuropeptide Y) (Brown et al., 1999).

Starved male flies court less, and starved female flies are less receptive. In females, this effect is partly mediated through an interaction between a food odor and a sex pheromone (Lebreton et al., 2015). The DA1 glomerulus exhibits a strong response to cVA (Datta et al., 2008), while it exhibits no significant response to the smell of vinegar. However, the smell of vinegar has a strong effect on the response of DA1 to cVA in females in a manner dependent on hunger status such that the response of DA1 to cVA is significantly lower in a starved female that is exposed to the smell of vinegar. This result is consistent with starved females being less responsive to male courtship when food is available (Lebreton et al., 2015). This work suggests that insulin signaling partly controls the adjustment of cVA attraction according to nutritional state. Interestingly, a recent study (Zhang et al., 2022) revealed that sugar intake-induced insulin signaling can reduce male mating motivation through the activation of insulin receptors in P1 cells, which serve as the central hub that controls male courtship, integrating both internal and external information.

In another study exploring the effects of food availability on mating behaviors, Grosjean et al. (2011) established that some food odors enhance the sexual behavior of *Drosophila melanogaster* males. Silencing of *fru* + chemosensory Ionotropic glutamate receptor 84a (IR84a) expressing ORNs caused a dramatic reduction in male courtship activity. IR84a-expressing neurons are activated not by fly-derived chemicals but by the aromatic odors phenylacetic acid and phenylacetaldehyde, which are widely found in fruit and other plant tissues. Projection neurons downstream of the IR84a ORNs converge in the pheromone processing region of the lateral horn, possibly promoting male courtship behavior by enhancing the response to pheromones secreted by the female, although this was not shown directly in this study. While the olfactory responses of IR84a ORNs are not significantly different between sexes, *Ir84a* mutant females do not show overt defects in reproductive behaviors, including in copulation latency, success, or duration (Grosjean et al., 2011). While work published by Lebreton et al. (2015) found that food-derived cues can affect female receptivity and first-order pheromone processing, Grosjean et al. (2011) reported that food-derived cues affected only male mating decisions, likely though the effect of food-derived cues on high order pheromone processing. The effect of food odorants on female receptivity therefore seems less robust. It is possible that the effect of nutritional state and available food on female receptivity depends on her mating state. Female flies are known to mate up to six times in nature (Markow, 2011) and may lay up to 80 eggs per day (Cohet and David, 1978). It has been reported that until females have used most of their stored sperm, remating is less likely to occur when food was absent or contact with food was prevented. Food availability, however, has little effect on the incidence of remating once stored sperm was depleted and no effect on initial virgin mating frequency (Harshman et al., 1988). One possible interpretation of these observations is that while virgin females accept copulation attempts from courting males even in the absence of food-derived sensory cues, the remating probability is modulated by these environmental cues (Chapman and Partridge, 1996; Billeter and Wolfner, 2018). This could be explained by a model wherein mating has costs for the female (Wigby and Chapman, 2005; Barnes et al., 2008), while the potential benefit of remating is lower than that of the initial mating.

*Drosophila* males that are deprived of access to both food and sex have competing needs. Choosing between feeding and mating is modulated by food quality and internal drive. Two mechanisms were recently proposed to govern this choice between feeding and mating in starved males. In the first, feeding-promoting tyramine receptor neurons and courtship-promoting P1 neurons are antagonistically modulated by tyramine and food detection (Cheriyamkunnel et al., 2021). In the second, a gut-derived, nutrient-specific neuropeptide hormone Dh31 propels a switch from feeding to courtship, where two distinct populations of Dh31 receptor neurons inhibit feeding and promote courtship through allatostatin-C and corazonin, respectively (Lin et al., 2022). How these two proposed mechanisms are related to each other, and what other signaling pathways are involved in feeding-mating balance control remains to be determined.

Lastly, it is worth noting that while nutritional state and food availability modulate fly mating decisions, as discussed above, the converse is also true, as female flies change their food preferences post-mating (Yang et al., 2008; Ribeiro and Dickson, 2010; Azanchi et al., 2013; Walker et al., 2015; Hussain et al., 2016). Therefore, feeding and mating circuits are bidirectionally linked.

## Discussion

In this review, we have explored how mating behaviors are flexibly controlled depending on both internal and external factors across two model systems—mice and flies, revealing that similar factors modulate mating behaviors in both species, potentially reflecting certain similarities with respect to the needs of these model organisms.

### Flexible mating behaviors in mice and flies

The exploration of the internal and external factors that modulate mating behaviors in mice and flies revealed that some factors are relevant in one species, but not in the other (Figure 1). For example, while the estrus cycle modulates mating behaviors in mice (see below), it does not exist in flies. Conversely, oviposition (egg laying)—a post-mating behavior—is relevant for flies, but not in mice. Memory over the span of weeks has very little ethological relevance in flies, while mice are likely less sensitive to temperature changes as compared to cold-blooded flies, and to what extent emotional states are relevant for different species remains a subject of debate (Adolphs and Anderson, 2018).

These unique factors that are known to contribute to the modulation of mating behaviors in only one species may be due to the differences in their biological/physical systems, ethological relevance, or a lack of literature focused on these specific questions. The estrus cycle has a major impact on female receptivity in rodents. Females in estrus display high sexual receptivity to male mating attempts, whereas females in diestrus show low sexual receptivity (Jennings and de Lecea, 2020). Recent studies have revealed some of the mechanisms whereby the hormonal states of females modulate their sexual receptivity and motivations. Inoue et al. (2019) demonstrated that periodic changes in the connectivity between certain neural circuits regulate the estrus cycle-dependent sexual receptivity. Progesterone receptor positive VMHvl (VMHvl^PR^) neurons project to the AVPV and this projection is necessary to display sexual receptivity in OVX plus hormone-primed (estrus) females. The presynaptic terminal density of VMHvl^PR^ neurons in the AVPV and functional connectivity between VMHvl^PR^ neurons and AVPV neurons are three times higher in OVX plus hormone-primed females relative to OVX females (diestrus). This is an interesting example of how the modulation of mating behavior circuits can also occur through the periodic anatomical remodeling of circuits, rather than just as a result of the modulation of excitation/inhibition balance in an anatomically stable circuit. Another example of the mouse-specific modulation of mating behavior relates to the ability to recognize an individual partner. Specifically, the motivation of the male to remate is higher when the prospective female is a novel female as compared to remating with the same female. While it is possible that flies are capable of distinguishing between individuals (Schneider et al., 2018), no effect of fly identity on remating has to date been demonstrated. This difference could reflect a gap in the literature or a real biological difference.

Despite their phylogenetic distance, mating behaviors in mice and flies seem to be affected by multiple common factors, often in comparable ways. Social hierarchy (losers/winners) reportedly has a stronger impact on males relative to females in both mice and flies, with the winners being more successful in mating in both organisms. Group-rearing, as compared to rearing in isolation, has profound effects on the circuits controlling mating behaviors in both species, from sensory processing to behavioral control. For example, social experience has been shown to modulate the responses of female mice to urine odors and the responses of female flies to male courtship song. Strikingly, there is evidence that social and sexual experience may overcome even severe circuit manipulations: While disruption of either the olfactory or vomeronasal system can have a strong adverse impact on male sexual behavior in many rodents, these effects are less severe if subjects are sexually experienced when they undergo disruptive manipulation. In *Drosophila* species, males that harbor mutations in the sex determination gene *fru* do not court, while mutant males that are housed with other males do court. In both species, there is thus evidence that even limited social experience may modulate the wiring of some neural circuits.

### The neural basis of flexible mating behaviors

Circuit nodes controlling mating behaviors can be broadly divided into three levels: sensory processing, behavioral decision making, and motor execution. As is evident from the studies of flies and mice reviewed above, internal and external factors influence neural responses at all three levels. While a simplified feed-forward model of information flow from the sensory periphery to the motor periphery is often used (as in Figure 2), the cells and brain structures that are involved in controlling mating behaviors in both mice and flies are highly interconnected (Auer and Benton, 2016; Wei et al., 2021). Moreover, recurrent connectivity may play a role in controlling persistent brain states, as was recently shown both in flies (Zhao et al., 2018; Zhang et al., 2019; Deutsch et al., 2020) and mice (Kennedy et al., 2020). Critically, while short (sub-second or a few seconds long) persistent activity has been intensively studied in the context of working memory (Major and Tank, 2004; Aksay et al., 2007; Barak and Tsodyks, 2007; Zylberberg and Strowbridge, 2017), far less is known about the neural basis for persistent states, such as emotional or motivational states, which typically modulate animal behaviors for many seconds to minutes. Computational modeling suggests that persistent activity that maintains stimulus identity (e.g., the existence of a rival or a predator) requires both recurrent excitation and slow-acting neuromodulation (Kennedy et al., 2020), although more experimental evidence is needed to support this idea.

Hormones and neuromodulators seem to play pivotal roles in the flexibility of mating behavior, though likely to a greater extent in rodents than in flies. DA (and to a lesser extent, serotonin) has been reported to be a currency of sexual motivation in both species, where DA levels in specific circuits link recent mating experiences with sexual satiety/motivation. In both species (and in both sexes) low nutritional levels reduce mating motivation. Relatively small populations of neurons (MPOA and VMHvl ERα neurons in mice, pC1 in flies) integrate multisensory information and control flexible mating behaviors in these two species. As previously noted, these similarities may be superficial and coincidental, or may represent conserved or analogous modules for the high-level control of flexible mating behaviors in both model systems. Another plausible mechanism for incorporating direct mating cues regarding a potential mating prospect and other internal or external factors is a balance between inhibition and excitation that inputs to a common node. In flies, for example, inhibitory and excitatory pathways converge on the command neuron P1. P1 neurons in male flies control the initiation and persistence of courtship, and the balance between the excitatory and inhibitory inputs to this population determines the level of P1 activity. These inputs to P1 carry both pheromonal and olfactory information about the prospective mate, leading to a higher net response of P1 to an appropriate mating partner. It is tempting to speculate that a balance between inhibitory and excitatory inputs may also serve to integrate information about the environment (e.g., the existence of predators, rivals, or unhealthy food sources) and about a mating prospect (e.g., its song quality or size), thus allowing for flexible mating decisions. NPY and its fly homolog NPF are associated with both feeding and mating motivation, possibly linking the two behaviors.

### Translational relevance

Mating behavior is fundamental and universal among all species, including humans. Based on the assumption that mating is a primitive behavior that is essential for the survival of the species, the control of mating behavior may be relatively preserved over the course of evolution. Efforts should be made to leverage the knowledge gained from animal studies to shed more light on human sexual behavior and the underlying neural circuitry with the goal of answering questions including: How do social isolation, sexual experience, and social context modulate sexual behaviors in healthy and non-healthy human individuals? How is sexual behavior modulated by internal motivational states? What goes wrong in this system in the case of pathological behaviors?

A better understanding of the relationship between the mechanisms governing flexible sexual behaviors and non-adaptive or pathological behaviors is missing in both clinical and non-clinical human populations. Particularly in disorders characterized by rigid and/or repetitive behaviors, such as obsessive-compulsive disorder or autistic spectrum disorders, social challenges are common and often hamper sexual relationships (Gordon, 2002; Kellaher, 2015). In these disorders, inflexible social behaviors may expose affected individuals to dysfunctional interpersonal contexts. Even subtle limitations in the capability to integrate contextual environmental factors together with innate factors in the context of social and sexual behavior may result in significant dysfunction of marital and social relationships. Furthermore, overly flexible and unstable sexual behavior, as frequently observed in borderline personality disorder (de Aquino Ferreira et al., 2018), also plays a pivotal role in this type of psychopathology.

What is the neural basis of maladaptive sexual behaviors? The dysfunction of sexual behavior can arise at different stages of the sensorimotor pathway. For example, individuals that experience compulsive sexual behavior (CSB) have been reported to exhibit enhanced attentional bias to explicit cues (Mechelmans et al., 2014), possibly paralleling the observations that addiction disorders are characterized by biases in selective attention toward drug cues (e.g., Ersche et al., 2010; Cousijn et al., 2013). Understanding the basic mechanisms underlying flexible sexual behaviors in model organisms will help to elucidate these mechanisms in human individuals under both physiological and pathological conditions.

### Future directions

More work is needed to reveal how different nodes in the sensorimotor pathway are modulated by different internal and external factors and over different timescales. For example, it remains to be established whether specific internal or external factors are more likely to modulate the periphery or the higher order centers and whether different mechanisms (e.g., neuromodulation or rewiring) are more likely to occur in response to different factors or at different levels of the circuitry. More broadly, it also remains uncertain whether there are any unifying principles for the control of flexible mating behaviors across taxa. Different conceptual models have been suggested for the neural circuit mechanisms that encode motivational states. According to Tinbergen’s hierarchical model for behavioral decisions (Tinbergen, 1951), the higher order command (or “apex”) neurons receive input from the environment, internal states, and prior experiences. Based on this information, these apex neurons choose between different second-level downstream behavioral outputs, which tend to inhibit one another. These second-level nodes can further trigger specific aspects of the behavior through third-order neurons. A somewhat different view is that reproductive circuits, much like feeding circuits, are under homeostatic control *via* a closed loop system that computes the difference between the animal’s need and the current state at any given point in time (Lee and Wu, 2020). Uncertainty also remains regarding how internal factors such as satiety, hunger, or sexual maturation fit into such conceptual models in the context of mating behavior and which nodes are modulated by previous experience. While some answers to these questions are emerging in the literature, as discussed above, many of these questions still remain open.

In both rodents and flies, there have been significantly fewer studies addressing behaviors and neural circuits in females relative to the numbers performed in males. There are a few reasons for this bias. First, as in many species, including mice and flies, the males actively court and the females are considered more passive, such that quantifying mating phenotypes in males (e.g., singing or licking in flies, singing, investigation, and mounting in mice) is easier. Second, in some species, including mice, the female estrous cycle is considered to add an experimental factor that must be controlled for, whereas this can be avoided by using males as experimental subjects. As the brain, body, and associated physiology are subject to sexual dimorphism, the knowledge derived from studies of males cannot simply be applied to females, and therefore more research is necessary to comprehensively understand the biology of mating behaviors and reproduction. While significant progress has been made in dissecting the neural circuitry underlying mating behaviors in female mice and flies (Ellendersen and von Philipsborn, 2017; Inoue et al., 2019; Deutsch et al., 2020; Liu et al., 2022), this gap remains to be closed.

While using genetic models such as mice and flies to study the neural basis of behavior has clear advantages, a comparative approach involving a wider range of animal models is critical if we aim to understand how nervous systems have developed to enable adaptive mating behaviors in different species and under different evolutionary constraints (Yartsev, 2017). Moreover, laboratory animals were selected over generations for phenotypes that fit the needs of experimentalists, and this selection may have a profound effect on social behaviors in both males and females. For example, female aggression is more profound in wild-caught mice than in laboratory mice (see e.g., Been et al., 2019). Emerging tools now make it possible to manipulate neural circuits in non-genetic model systems (Ding et al., 2019; Navabpour et al., 2020). This will likely lead to more comparative studies in diverse laboratory and wild-type animal models.

As mating behaviors are critical for animal survival, they must have a pre-programmed component, but at the same time, they must be flexible enough to allow the circuits to adapt to changing needs and environments. While much progress has been made in our understanding of the neural basis of mating control in genetic model systems including mice and flies, many questions remain open. We believe that the emergence of new experimental and computational tools, the study of males and females of diverse model species, and new theoretical models that take into account the broad ethological context of mating behaviors will ultimately lead to exciting new findings in this fundamental field of neurobiology in the near future.

## Author contributions

Both authors listed have made a substantial, direct, and intellectual contribution to the work, and approved it for publication.
